# Ultrasound-Guided Multiple Peripheral Nerve Blocks in a Superobese Patient

**DOI:** 10.1155/2014/896914

**Published:** 2014-01-22

**Authors:** Alper Kilicaslan, Ahmet Topal, Atilla Erol, Hale Borazan, Onur Bilge, Seref Otelcioglu

**Affiliations:** ^1^Department of Anaesthesiology, Meram Medical Faculty, Necmettin Erbakan University, 42080 Konya, Turkey; ^2^Department of Orthopaedic Surgery, Meram Medical Faculty, Necmettin Erbakan University, 42080 Konya, Turkey

## Abstract

The number of obese patients has increased dramatically worldwide. Morbid obesity is associated with an increased incidence of medical comorbidities and restricts the application choices in anesthesiology. We report a successfully performed combined ultrasound-guided blockade of the femoral, tibial, and common peroneal nerve in a superobese patient. We present a case report of a 31-year-old, ASA-PS II, super obese man (190 kg, 180 cm, BMI: 58 kg/m^2^) admitted to the emergency department with a type II segmental tibia shaft fracture and ankle dislocation after a vehicle accident. After two failed spinal anesthesia attempts, we decided to apply a femoral block combined with a sciatic block. Femoral blocks were successfully performed with US guided in-plane technique. Separate blocks of the tibial and common peroneal nerves were planned after the sciatic nerve could not be located due to the thick subcutaneous tissue. We performed a tibial nerve block at 2 cm above the popliteal crease and common peroneal nerve at the level of the fibular head with US guided in-plane technique. The blocks were successful and no block-related complications were noted. Ultrasound guidance allows new approaches for multiple peripheral nerve blocks with low local anesthetic doses in obese patients.

## 1. Introduction

The number of obese patients is gradually increasing worldwide. The World Health Organization estimates that, by 2015, there will be 2.3 billion overweight (BMI 25–30 kg/m^2^) and 700 million obese (BMI > 30 kg/m^2^) adults worldwide [1]. Anatomic and physiological alterations occur in association with obesity, particularly in the airway and in the cardiovascular, respiratory, gastrointestinal, and neurological organ systems. These changes increase the incidence of comorbidities and cause limitations and problems in anesthesiology procedures [2].

For obese patients, regional anesthesia provides many advantages compared to general anesthesia, such as avoiding airway manipulation and systemic effects of anesthetic agents, and provides better postoperative pain control [3]. However, the failure rate increases in regional anesthesia procedures performed in obese patients due to the increased depth of nerve structures, the disappearance of landmarks, and difficulties in positioning [4].

On the other hand, the increase in the use of ultrasonography in recent years eliminates many limitations. Ultrasonography enables direct visualization of nerve structures, reduction in complications, and identification of new peripheral nerve block approaches [5].

In this study, we aimed to present the anesthesia management of a superobese patient who underwent ultrasound-guided multiple peripheral nerve blocks (the femoral, tibial, and common peroneal nerves) following the failure of spinal anesthesia.

## 2. Case Report

A 31-year-old, ASA II, superobese male patient (190 kg, 180 cm, BMI: 58 kg/m^2^) was admitted to the emergency department with a segmental Gustilo-Anderson type IIIA open tibial fracture and ankle dislocation following an in-vehicle traffic accident. The initial proper management of open fracture and joint dislocation was performed by orthopaedic and traumatology surgeons in the emergency department. The patient had no history of additional diseases. The preoperative airway examination revealed a class 3 Mallampati airway and, in light of the difficulty of intubation, an intrathecal block with a 150 mm needle was planned. After the written consent was obtained, 2 mg IV midazolam and 50 *μ*g fentanyl were administered for sedation. The vertebral anatomical structures were barely distinguishable despite ultrasound guidance. After two unsuccessful attempts, we decided not to use spinal anesthesia and planned to perform a combined sciatic and femoral nerve block. As a tourniquet was not required above the knee, the blockade of these two nerves would be adequate for surgical anesthesia.

**Figure 1:**
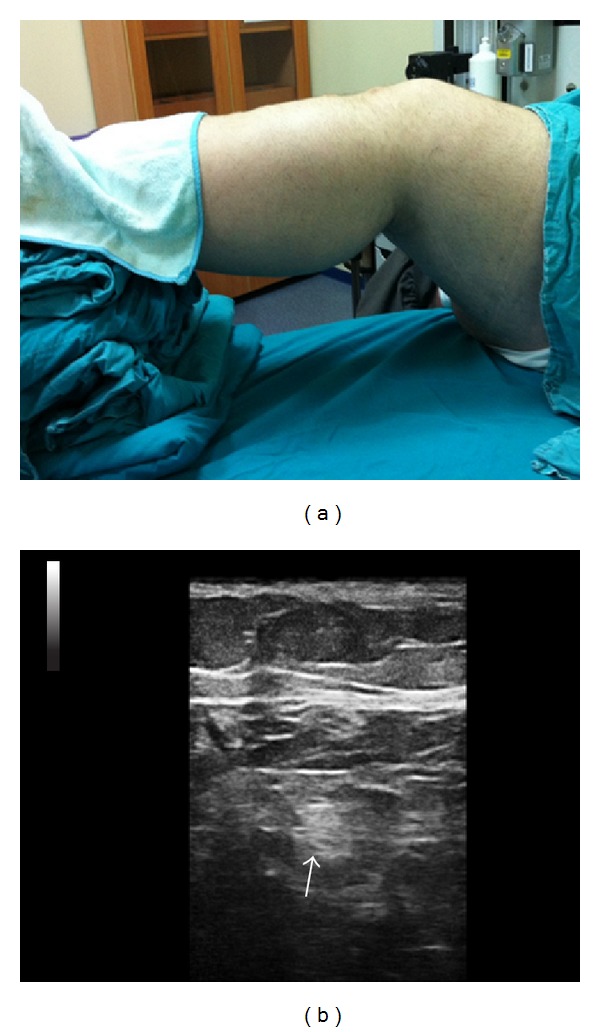
(a) Positioning for popliteal approach to the tibial nerve block. (b) Transverse sonogram in the popliteal region showing the tibial nerve as a hyperechoic nodule (arrow).

**Figure 2:**
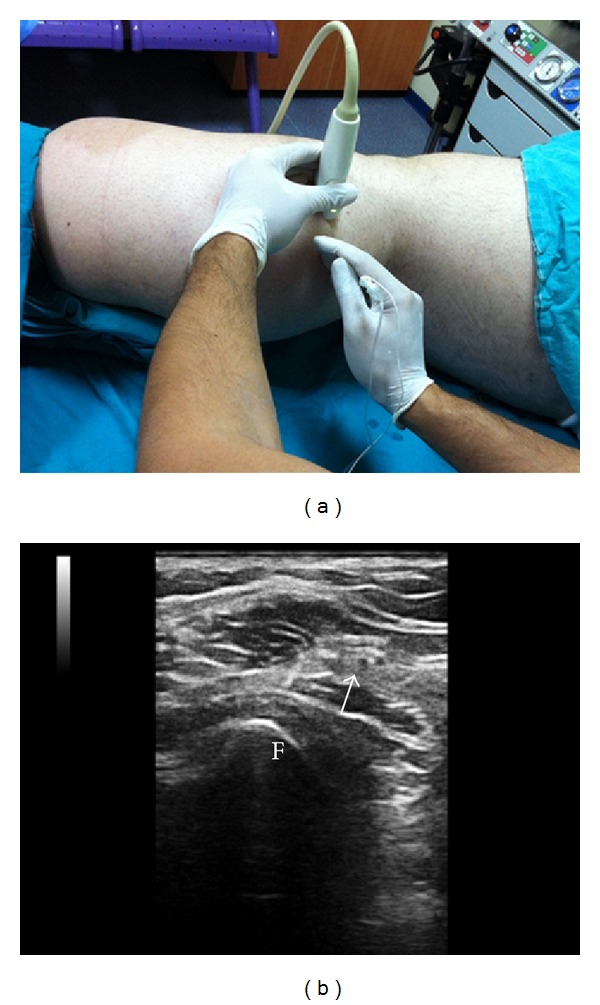
(a) Ultrasound probe placement for common peroneal nerve block at the head of the fibula. (b) Sonogram of the common peroneal nerve at the head of the fibula (arrow). F: fibula.

Before the femoral nerve block, the pannus was taped cephalad as the fatty tissue hanging from the abdomen made it difficult to access the inguinal region. The femoral nerve was identified at a depth of 5 cm with a linear US probe (Esaote, 10–18 MHz, Florence, Italy). Fifteen mL (8 mL of 0.5% levobupivacaine and 7 mL of 2% lidocaine) of local anesthetic mixture was administered around the femoral nerve using the in-plane approach. Due to the difficulties in situating the patient in the prone position, the distal extremities of the patient were elevated with folded blankets in the supine position (Figure 1(a)). The sciatic nerve could not be visualized despite several attempts using both linear and convex probes; therefore separate blocks of the tibial and common peroneal nerves were planned. The tibial nerve was identified in the popliteal fold, posterior to the popliteal artery at a depth of 3 cm (Figure 1(b)). Visualization of the tibial nerve was highly difficult in areas more proximal than the point 2 cm above the popliteal fossa. The common peroneal nerve (CPN) and the bifurcation of the sciatic nerve could not be visualized despite several attempts using both linear and convex probes. Therefore, 10 mL (7 mL of 0.5% levobupivacaine and 3 mL of 2% lidocaine) of local anesthetic mixture was administered around the tibial nerve 2 cm above the popliteal fold by using a linear probe. Screening was performed in the distal direction over the lateral side of the patient's leg with the linear probe placed transversely on the edge of the patella to identify the CPN (Figure 2(a)). The nerve was visualized posteriorly and laterally to the fibular head, 4 cm distally to the edge of the patella at a depth of 2 cm (Figure 2(b)). At this level, 10 mL (7 mL of 0.5% levobupivacaine and 3 mL of 2% lidocaine) of local anesthetic mixture was administered around the nerve with the in-plane technique using a linear probe. A 100 mm 21-G echogenic needle (Pajunk, Geisingen, Germany) was used for all blocks. A nerve stimulator was not used for any blocks since the patient had extremity pain associated with trauma. Surgical anesthesia was established within 30 minutes after anesthetic administration and no complications occurred in association with the blocks. Emergent reduction and fixation with an external fixator were performed for the ankle dislocation and fracture of the tibia. Additional local anesthetic injection or additional sedation was not required during the operation. The operation lasted for approximately 1.5 hours and was completed without problems. The patient's consent was obtained for the publication of this case report.

## 3. Discussion

The likelihood of the presence of medical comorbidities such as hypertension, cardiopulmonary disease, type 2 diabetes, obstructive sleep apnea and venous thromboembolism increases in superobese (BMI ≥ 50) patients [6]. Obesity is associated with increased perioperative risks of difficult airway, cardiopulmonary dysfunction, acid aspiration, and mortality. The use of regional anesthesia instead of general anesthesia in obese patients decreases these risks and additionally it provides safe and effective postoperative analgesia [7, 8]. Unfortunately, our attempt at spinal anesthesia failed in this case despite ultrasound guidance. In the application of neuraxial blockade on obese patients, palpation of bony landmarks and the identification of the midline are more difficult, and the presence of fat packets may cause false-positive loss of resistance during the advancement of the needle. The identification of the intervertebral structures using ultrasonography is even more difficult in these patients [3]. Cases with failure and complications were reported even with the use of ultrasonography during epidural anesthesia [9]. Additionally, the decrease in the epidural space volume in obese patients leads to an unpredictable spread of local anesthetics and variable block levels. The risk of cardiopulmonary collapse and respiratory problems associated with increased block levels is higher in obese patients [7].

Despite the technical difficulties, peripheral nerve blocks may help to reduce these problems. Peripheral nerves are under dense adipose tissues and located more deeply in obese patients. During the use of ultrasonography, the needles should be positioned at a more vertical angle since the target tissue is more deeply located and thus the visualization of the needle tip is more difficult. Therefore, more experience and expertise is required for the use of ultrasonography in obese patients [10]. The increase in adipose tissue leads to alteration of the sonoanatomy learnt for normal-weight individuals and makes it more difficult to identify the target structures; therefore we may need to try new approaches by changing our perspectives.

The CPN block is uncommon in clinical practice. The CPN passes around the fibular head laterally after passing through the popliteal fossa. The block of the CPN in this region was described by Ting et al. [11]. This level is ideal for visualization with US, since the nerve is superficially located and the fibular head can be used as a sonoanatomical landmark. The absence of major vasculature at this level is another advantage, since the popliteal artery passes medially below the knee. On the other hand, the proximity of the CPN to the fibular head in this region increases the risk of injury associated with compression [12]. Since there is inadequate data on the safety of this uncommon approach, the local anesthetic was limited to a volume of 10 mL in order to minimize the risk of nerve compression; no complications were observed following the block.

The curvilinear probe might be an appropriate option for obese patients because it uses lower frequencies and has improved penetration [13]. However, we do not have curvilinear probes in our department so we generally use linear probe for peripheral nerve blocks.

In conclusion, in the case of this superobese patient with difficult airway revealed by the preoperative examination, ultrasound-guided multiple peripheral nerve blocks were successfully performed following unsuccessful attempts for spinal block. Ultrasonography enables direct visualization of the nerves and reduction in the required local anesthetic doses and thus allows for multiple nerve blockade. In addition, ultrasound guidance enables the use of new peripheral nerve block approaches in obese patients. Due to these reasons, ultrasound-guided peripheral nerve blocks may be a good alternative to general anesthesia and central blocks for extremity surgeries in obese patients despite their technical difficulties. Based on our experience, the identification of more distal and superficial block points by individualized scanning instead of using common regions may be useful in the identification of physiological changes induced by obesity. Further studies are needed for the optimization of nerve block techniques and local anesthetic doses in obese patients.
